# Suppression of UCP2 alleviates leukemogenesis by enhancing branched-chain amino acids-induced oxidative stress via activating the PI3K/AKT/mTOR signaling pathway

**DOI:** 10.1016/j.gendis.2025.101794

**Published:** 2025-08-05

**Authors:** Agida Okohi Innocent, Yajie Shen, Yixuan Gao, Ruixin Sun, Kasimujiang aximujiang, Zizhen Xu, Jinke Cheng, Jiao Ma

**Affiliations:** a Department of Biochemistry and Molecular Cell Biology, Shanghai Jiao Tong University School of Medicine, Shanghai 200025, China; b State Key Laboratory of Genetic Engineering, School of Life Sciences and Huashan Hospital, Shanghai Engineering Research Center of Industrial Microorganisms, Fudan University, Shanghai 200438, China; c Department of Biochemistry and Molecular Biology, School of Basic Medical Sciences, Xinjiang Medical University, Urumqi, Xinjiang 710061, China; d Department of Laboratory Medicine, College of Health Science and Technology, Ruijin Hospital, Shanghai Jiao Tong University School of Medicine, Shanghai 200025, China

**Keywords:** AML branched-chain amino acids, Leukemogenesis, Oxidative stress, PI3K/AKT/mTORsignaling, UCP2

## Abstract

Although the cellular role of uncoupling protein 2 (UCP2) in tumorigenesis has been reported in various solid tumor models, its role in leukemogenesis remains elusive. Herein, we demonstrated that UCP2 was highly expressed in AML and significantly associated with poor prognosis and chemoresistance, suggesting that UCP2 can be used as a potential biomarker in acute myeloid leukemia. Mechanistically, *in vitro* and *in vivo* silencing of UCP2 significantly impairs acute myeloid leukemia cell growth and survival, accompanied by the disruption of mitochondrial homeostasis. Interestingly, RNA-sequencing analysis and metabolic mass spectrometry revealed that silencing UCP2 resulted in accumulated branched-chain amino acids (BCAAs), which induced oxidative stress through the PI3K/AKT/mTOR signaling pathway. Additionally, the lack of BCAAs restored leukemic cell growth and survival and decreased mitochondrial ROS production induced by inhibiting UCP2. More importantly, supplementation of BCAA enhanced the anti-tumor activity of genipin, a selective inhibitor that targets UCP2, resulting in significantly reduced acute myeloid leukemia blasts, increased mouse survival, and magnified oxidative stress. Taken together, our study elucidates the rationale of targeting the UCP2-BCAA-PI3K/AKT/mTOR signaling axis in leukemogenesis and provides a novel strategy for leveraging the metabolic dependencies of leukemic cells.

## Introduction

Acute myeloid leukemia (AML) is a multifaceted and devastating group of blood cancers and accounts for a staggering 80% of acute leukemia cases in adults.^1^ The incidence of AML has witnessed a disturbing upward trend, skyrocketing from 79,372 cases in 1990 to 144,645 in 2021.^2^ This surge underscores the need for enhanced awareness, research, and therapeutic interventions.^3^ Despite the improved clinical outcome of targeted therapy or immunotherapy,^4^^,^^5^ the frequent occurrence of genetic mutations,^6^ epigenetic changes,^7^ and mitochondrial dysregulation^8^ is still troublesome. This disease is highly heterogenicity and attributed to poor prognosis and high relapse rate in response to the standard therapy such as “3 + 7”,^9^ which emphasizes the urgent need for developing novel therapeutic strategies to improve the AML clinical outcome.

Uncoupling protein 2 (UCP2) has been shown to regulate mitochondrial biogenesis,^10^ metabolism, and reactive oxygen species (ROS) production. Mechanistically, UCP2 activity dissipates the mitochondrial membrane potential (ΔΨm),^11^ resulting in decreased ATP production and altered cellular bioenergetic metabolism.^12^ The proton gradient across the inner mitochondrial membrane fuels mitochondrial ROS production^10^; however, this gradient can be regulated by UCP2^13^ in the mitochondria, as the mitochondria are primarily known for generating ATP under normal oxygen levels.^14^ Pathologically, UCP2 facilitates tumor cell growth and metastasis and promotes tumor cell survival in various tumor types such as non-small cell lung cancer, ovarian cancer, and cervical cancer.^15^, ^16^, ^17^ Despite the well-established oncogenic roles of UCP2 via regulation of mitochondrial reprogramming, its role in hematological malignancy is not well understood.

Leucine, isoleucine, and valine are three essential branched amino acids and account for approximately 20% of total protein intake in the human diet.^18^^,^^19^ After food consumption, digested branched-chain amino acids (BCAAs) were passively transported through the intestinal lumen with the assistance of Na^+^-dependent cotransporter L-type amino-acid transporter 1 (LAT1) and its heterodimer complex proteins.^20^^,^^21^ It is well known that BCAA plays a pivotal role in sustaining cellular homeostasis through energy balance, nutrient signaling, and nitrogen regulation.^22^ BCAA also has been implicated in regulating the pathogenesis of metabolic disturbance-related diseases, such as type II diabetes, cardiovascular disease, non-alcoholic liver disease, and cancers.^23^, ^24^, ^25^, ^26^, ^27^ However, in the context of cancer, elevated BCAA does not always supply the glucose for tumor cells to grow, accelerate malignant transformations, and maintain tumor cell stemness, but has also been linked to inhibited tumor growth, suggesting its potential anti-tumor properties.^28^

In the current study, we have elucidated the pathogenetic role of UCP2-modulated BCAAs in leukemogenesis via regulating the phosphoinositide 3-kinase (PI3K)/protein kinase B (AKT)/mammalian target of rapamycin (mTOR) signaling axis.

## Materials and methods

### Cell lines, primary cells, and culture conditions

The AML cell lines Kasumi-1, MOLM-13, KG-1, and THP-1 were cultured in RPMI 1640 supplemented with 10%–20% fetal bovine serum and 1% penicillin/streptomycin. Kasumi-1 (CLR-2724), KG-1 (CRL-8031), and THP-1 (TIB-202) were purchased from ATCC, and MOLM-13 (ACC-554) was purchased from DSMZ-German Collection of Microorganisms and Cell Cultures GmbH. All cells were routinely checked for mycoplasma infection using the MycGuard Plus-Color One-Step Mycoplasma Detection Kit (Cat. No. 40612ES25, Yeasen), and maintained in a humidified 37 °C/5% CO_2_ incubator. To explore whether the lack of BCAAs counteracted the silencing of UCP2-induced phenotypes, THP-1 or KG-1 cells were sub-cultured in either normal RPMI 1640 or RPMI 1640 without BCAAs (Cat. No. R7130, Sigma) before harvesting cells for subsequent experiments.

### Compounds and antibodies

Genipin (Cat. No. HY-17389), l-leucine (Cat. No. HY-N0486), l-isoleucine (Cat. No. HY-N0771), and l-valine (Cat. No. HY-N0717) were supplied by MCE China (Shanghai, China). All compounds, except for those used in *in vivo* studies, were reconstituted in dimethylsulfoxide, and the compounds were stored at 100 mM stock concentrations at −80 °C and used at the indicated doses suggested by the vendor. Immunoblotting antibodies, including phospho-Akt (Ser473) antibody (Cat. No. 9271S), Akt (pan) (11E7) rabbit mAb (Cat. No. 4685), phospho-p44/42 MAPK (Erk1/2) (Thr202/Tyr204) (D13.14.4E) XP rabbit mAb (Cat. No. 4730S), p44/42 MAPK (Erk1/2) (137F5) rabbit mAb (Cat. No. 4695), mTOR (7C10) rabbit mAb (Cat. No. 2983S), and phospho-p70 S6 kinase (Thr389) (108D2) rabbit mAb (Cat. No. 9234S), were purchased from Cell Signaling Technology (Danvers, USA). Flow cytometry antibodies, including phycoerythrin/cyanine7 anti-mouse CD34 antibody (Cat. No. 128617), APC anti-human CD45 recombinant antibody (Cat. No. 384403), and Pacific Blue anti-mouse CD38 antibody (Cat. No. 102719), were purchased from BioLegend (San Diego, USA). The UCP2 antibody (Cat. No. 11081-1-AP) was purchased from Proteintech (Rosemont, USA), and Complex III was purchased from Santa Cruz Biotechnology (Dallas, Texas, USA).

### Establishment of UCP2 stable transfectants

AML cells were infected with pooled UCP2 shRNA lentivirus (Applied Biological Materials, Shanghai, China). Seventy-two hours post-infection, positive cells were selected using a flow cytometry system. Western blotting analysis was performed to identify UCP2 knockdown clones. Clones infected with scrambled shRNA lentivirus were selected and used as controls.

### RNA extraction and PCR

Total RNA was isolated using TRIzol (Invitrogen) and transcribed into cDNA using the High-Capacity cDNA Reverse Transcription Kit (Applied Biosystems, Foster City, CA, USA) according to the manufacturer’s instructions. After adding the Cyber-Green PCR Master Mix, quantitative real-time PCR was performed using the ABI 7500 Real-Time PCR System (Applied Biosystems). Experiments were repeated three times. Relative expression levels were determined using the 2-Ct method.

### Metabolic mass spectrometry assay

Twenty microliters of supernatant were chromatographed using an Agilent 1290 UHPLC system equipped with a 2.1∗100 mm, 1.8 μM ACQUITY UPLC HSS T3 column (Waters, Milford, Massachusetts, USA) at a flow rate of 0.5 mL/min. Solvent A (10 mM NH_4_Ac in H_2_O) was held at 98% for 48 s, and then a linear gradient to 20% solvent B (ACN) was applied over the next 276 s. The column was held at 20% for 60 s and then equilibrated to 98% A for 216 s. The needle was washed before each injection with a mixed solvent (2% ACN in H_2_O). Multiple reaction monitoring was performed in positive ion mode using an AB Sciex 6500 plus QTRAP with the following transitions: *m*/*z* 380.1/233.3 for l-glutathione (L-GSH), and *m*/*z* 839.7/333.0 for lactyl-CoA, respectively.

### RNA transcription sequencing

RNA sequencing was conducted as described previously.^29^

### Cell proliferation assay

Two thousand viable AML cells were seeded in 96-well plates for the cell proliferation assay. The cell proliferation assay was performed using the flow cytometry live cell system. Cells were imaged every 24 h, 48 h, and 72 h, and the proliferation rates based on confluency were determined using flow cytometry.

### Metabolism assays

Nicotinamide adenine dinucleotide phosphate (NADP)/reduced NADP (NADPH), reduced glutathione/oxidized glutathione (GSH/GSSG), and glucose uptake assays (Abcam, Cambridge, UK; Promega, Madison, Wisconsin, USA) were carried out according to the manufacturer’s instructions.

### Detection of mitochondrial ROS

MitoSOX Red probe (Thermo Scientific Inc.) was used to detect mitochondrial ROS. Flow cytometry assays were performed according to the manufacturer’s instructions. Briefly, 1 million AML cells or primary AML cells were suspended in 0.5 mL RPMI-1640 medium containing 2.5 μM MitoSOX in a 37 °C water bath in the dark for 40 min. After washing with phosphate buffer saline (PBS), the red fluorescence was detected by FAC Scan using the LSRII SORP (Becton–Dickinson). Experiments were repeated three times.

### Detection of mitochondrial membrane potential (Δψm)

Ten thousand viable AML cells were seeded in 96-well plates and incubated overnight. Cells were incubated with a growth medium containing 2 μg/mL of JC-1 (Thermo Fisher, Waltham, Massachusetts, USA) for 30 min. The dye was removed, and the cells were washed with PBS. Fluorescence intensity was measured immediately by flow cytometry.

### Detection of mitochondrial mass

MitoTracker Red probe (Thermo Scientific Inc.) was used to detect mitochondrial mass. Flow cytometry assays were performed according to the manufacturer’s instructions. Briefly, 1 million AML cells or primary cells were suspended in 0.5 mL of RPMI 1640 medium. The cells were labelled with the desired reagents, such as antibodies and LIVE/DEAD Fixable Viability dyes. A 1X solution of reactive dye from the kit was prepared by adding 1 μL of reconstituted reactive dye per 1 mL PBS and well mixing. After washing with PBS and centrifugation at 1000*g* for 5 min, the supernatant was discarded, and the cells were further resuspended in 1 mL of 1X dye solution and incubated at the preferred temperature (37 °C is recommended) in the dark for 30–60 min, and further analyzed by flow cytometry using an appropriate excitation and emission channel.

### Detection of mitochondrial ATP

The amount of ATP was measured using the ATP assay kit (Beyotime, China). AML cells with UCP2 knockdown were collected and treated with 200 μL lysis buffer from the ATP detection kit. After centrifugation at 12,000 *g* at 4 °C for 5 min, supernatants were mixed with 100 μL ATP detection buffer. The optical density of the mixture was assayed with a luminometer (PerkinElmer). The concentration of ATP was calculated using a standard curve of ATP concentration versus absorbance.

### Bioinformatical analysis

The research on AML utilized datasets from The Cancer Genome Atlas (TCGA) and Genotype-Tissue Expression (GTEx) databases.^30^ The TCGA databases include 173 AML patient samples, and the GTEx databases include 70 healthy controls. To mitigate batch effects, the R language Combat package was used to merge the mRNA expression data from TCGA, which was subsequently converted from count values to transcripts per million (TPM) values. Acquisition of mitochondrial metabolism-related genes and identification of mitochondria-related hub genes for AML were performed using MitoCarta3.0 and clustering analysis with the “ConsensusClusterPlus” package in R. Pathways with Gene Ontology (GO) and Kyoto Encyclopedia of Genes and Genomes (KEGG) annotations, along with *p*-values below 0.05, and those enriched in other databases, were deemed significant.

### Identification of mitochondria-related hub genes for AML

MitoCarta3.0 is an inventory of 1136 human and 1140 mouse genes encoding proteins with strong support of mitochondrial localization, now with sub-mitochondrial compartment and pathway annotations. Using unsupervised clustering analysis with the “ConsensusClusterPlus” package in R, we identified clusters characterized by mitochondria-related features. We then proceeded to improve and validate a risk model built upon these hub genes related to mitochondrial metabolism. Initial screening using univariate COX analysis identified high-frequency genes potentially linked to prognosis. These candidates underwent further refinement through the LASSO regression method, which selects genes based on their expression levels (Least absolute shrinkage and selection operator regression (“glmnet” R package) was applied to select hypoxia-featured genes. By constructing a penalty function, the coefficients of the relatively unimportant variables can become zero and are eventually excluded from the model). A risk score formula was formulated using these selected genes, weighted by their respective LASSO regression coefficients. This risk score was determined for each individual in the training cohort, who were then categorized into groups by their median risk score into low-risk and high-risk categories following this specific format: Risk score.

Survival assessment utilized Kaplan–Meier curves, and comparisons between groups were conducted by applying the log-rank test to evaluate disparities in survival among the risk groups. The model’s ability to predict accurately was evaluated by means of a time-sensitive receiver operating characteristic curve assessment. Moreover, univariate and multivariate Cox regression assessments were conducted to confirm if the risk scores, alongside other clinicopathological factors, independently predicted prognosis. Survival assessment utilized Kaplan–Meier curves, and comparisons between groups were conducted by applying the log-rank test to evaluate disparities in survival among the risk groups. The model’s ability to predict accurately was evaluated by means of a time-sensitive receiver operating characteristic curve assessment. Moreover, univariate and multivariate Cox regression assessments were conducted to confirm if the risk scores, alongside other clinicopathological factors, independently predicted prognosis.

### Mouse model AML engraftments

Female NOD/SCID mice (5–6 weeks old) were obtained from Shanghai Ling Chang Biotechnology Co., China, with approval from Shanghai Jiao Tong University Medical School’s institutional animal facility. To study the impact of UCP2 on leukemic cells, we established AML xenotransplants by injecting 2 million UCP2 silencing stable transfectants into sub-lethally irradiated (2.5 Gy) mice via tail vein injection. Human AML engraftment was assessed 12 days post-transplantation. To further investigate the effects of the UCP2 selective inhibitor genipin on leukemogenesis, we established xenotransplants as described above. AML xenografts were treated with either PBS (vehicle control) or genipin via intraperitoneal injection. Mouse survival and tumor burden were monitored, with tumor burden assessed by flow cytometry. Following the sacrifice of mice, bone marrow mononuclear cells were harvested and subjected to immunostaining. The staining was performed at room temperature for 15 min. After washing, the cells were resuspended in FACS buffer containing 7AAD (diluted 1:20) and analyzed by flow cytometry to identify AML engraftment, characterized by the percentage of hCD45^+^ population.

### BCAA supplementation

AML xenografts were established as described previously. Mice were administered 25 mg/kg genipin at the indicated time interval and fed either a normal or high BCAA diet throughout the entire procedure. In the high BCAA-fed group, mice were fed with water containing 15 mg/mL valine, 15 mg/mL leucine, and 15 mg/mL isoleucine. Control mice (normal BCAA) were fed with normal sterile ddH_2_O. Blood was withdrawn from the back of the eye via the posterior ophthalmic venous plexus, and plasma BCAA levels were measured using a BCAA detection kit (Beyotime, China) according to the instruction manual.

### Statistical analysis

Statistical analysis was performed using the unpaired Student’s *t*-test to compare differences between two groups, and two-way ANOVA (Analysis of Variance) to compare differences among three or more groups. Linear regression analysis (GraphPad Prism version 6.01) was also used to examine correlations between primary AML cell viability and UCP2 expression levels, between primary AML cell viability and total ROS production, between primary AML cell viability and mitochondrial ROS production, and between mitochondrial ATP and mitochondrial membrane potentials. The survival analysis was performed using the log-rank (Mantel–Cox) test.

## Results

### Mitochondria-related genes were elevated in the high-risk AML group

**Figure 1:**
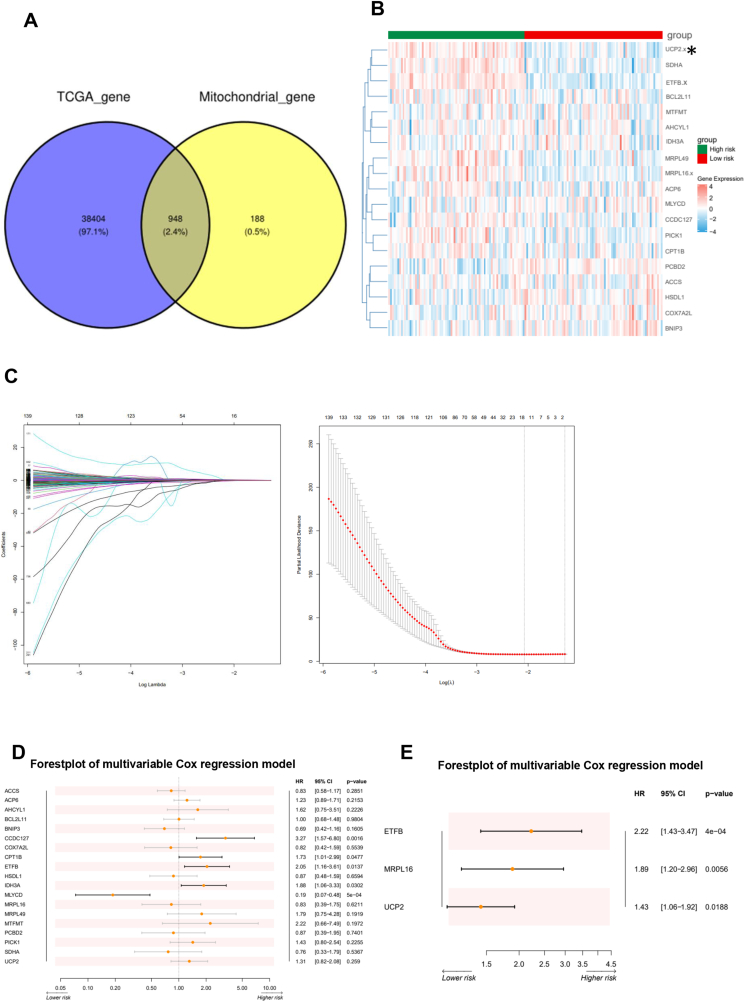
Mitochondria-related genes are significantly elevated in acute myeloid leukemia. **(A)** A Venn diagram shows the number of mitochondria-related genes that overlap between TGCA and mitochondria-related genes. **(B)** A heat map of the mitochondria gene expression profile in the low-risk and high-risk acute myeloid leukemia groups. **(C)** LASSO coefficient curves for mitochondria-related genes with non-zero coefficients determined by the optimal lambda. **(D, E)** Forest plot of multivariable and Cox regression model highlighting patients with UCP2 overexpression as high risk. UCP2, uncoupling protein 2.

Since AML is heterogeneous and targeting metabolism could be a potential therapeutic target in AML, we set out to screen the novel metabolism-related genes using bioinformatics. Venn diagram showed that the potential of an AML TCGA data set revealed 38,404 genes and 97.1% were elevated in the high-risk AML group, among which 188 genes were prognostic-related mitochondria genes, and 948 genes were overlapped between two sets (Fig. 1A). A heatmap showed that mitochondria-related genes were significantly up-regulated in the high-risk AML group compared with the low-risk AML group (Fig. 1B). Then, we integrated the disease status of AML, together with mitochondria-related genes, and performed regression analysis using the LASSO and multivariable Cox method, and screened out 19 genes that were potentially associated with AML prognosis (Fig. 1C–E). These data elucidate the potential therapeutic implications of mitochondrial metabolism in hematological malignancy.

### UCP2 acts as a potential prognostic biomarker in AML

**Figure 2:**
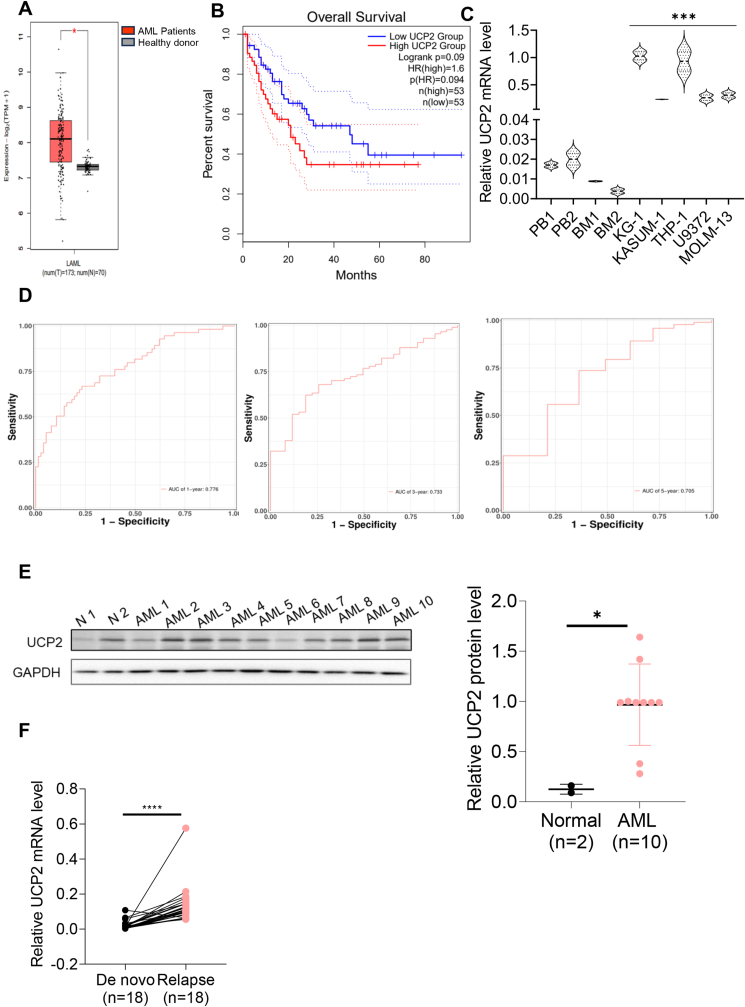
UCP2 acts as a potential prognostic marker in AML. **(A)** TGCA database analysis of UCP2 mRNA level in healthy donors identified in the gray box (*n* = 70) versus AML patients identified with the red box (*n* = 173). **(B)** Kaplan–Meier plot of UCP2 overall survival in the low-risk versus high-risk AML group. **(C)** Quantitative PCR of UCP2 mRNA level in a panel of AML cell lines versus either peripheral blood (PBL) or bone marrow (BM) normal controls. **(D)** Time-dependent receiver operating characteristic curve according to the 1-year, 3-year, and 5-year survival of the area under the curve (AUC value). **(E)** Immunoblotting of UCP2 protein level in a panel of AML primary cells (*n* = 10) versus healthy donors (*n* = 2). Band intensity was measured using the Image J software and normalized to GAPDH control. **(F)** Quantitative PCR of UCP2 mRNA level in *de novo* AML primary cells (*n* = 18) versus paired relapsed AML primary cells (*n* = 18). All experiments were repeated three times. Data represent mean ± standard deviation from technical triplicates (∗*p* < 0.01, ∗∗*p* < 0.05, and ∗∗∗*p* < 0.001; Two-way ANOVA for BM or PBL controls versus cell lines, unpaired *t*-test for the normal versus AML and the *de novo* versus the relapse). UCP2, uncoupling protein 2; AML, acute myeloid leukemia.

To explore whether UCP2 acted as a potential prognostic biomarker in AML, bioinformatics analysis of the mRNA level of UCP2 in an AML TGCA data set was conducted. The results showed that UCP2 mRNA level was highly expressed in AML patients (*n* = 173) compared with healthy donors (*n* = 70) (Fig. 2A). In addition, the Kaplan–Meier survival study in the TCGA data set showed that the UCP2 high-risk group exhibited shorter overall survival than the UCP2 low-risk group (Fig. 2B), indicating its potential prognostic role in diffuse large B-cell lymphoma pathogenesis. Moreover, UCP2 was highly expressed in a panel of AML cell lines (*n* = 5, *p* < 0.001) compared with either peripheral blood or bone marrow controls (Fig. 2C). In addition, the time-dependent receiver operating characteristic curve according to the 1-year, 3-year, and 5-year survival of the area under the curve further confirmed that UCP2 expression was potentially associated with the poor AML prognosis (Fig. 2D). However, Figure 2E demonstrated that UCP2 protein levels were elevated in primary AML patient samples (*p* < 0.05, *n* = 10) (see Table S1 for patients’ clinical information) compared with healthy donors (*n* = 2). Furthermore, UPC2 was highly expressed in 18 relapsed cases compared with the paired *de novo* AML primary cells (*p* < 0.001) (Fig. 2F; see Table S1 for patients’ clinical information), suggesting the potential prognostic role of UCP2 in AML.

### Silencing UCP2 inhibits leukemogenesis and disrupts mitochondrial homeostasis

**Figure 3:**
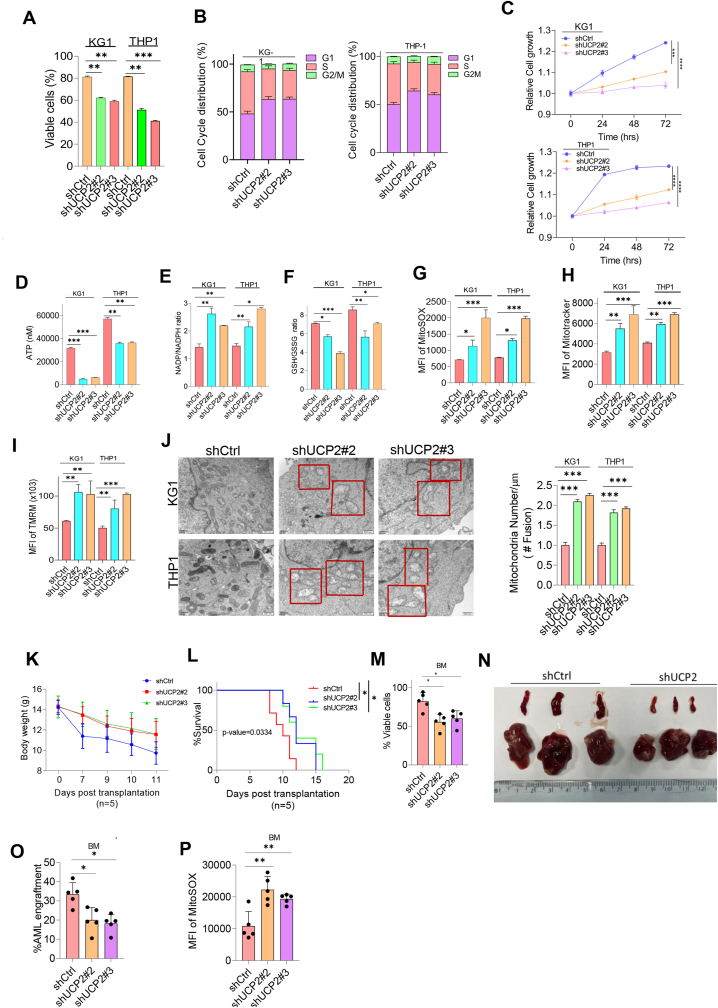
Silencing UCP2 inhibits leukemogenesis and disrupts mitochondrial homeostasis. THP-1 or KG-1 cells were transduced either with lentiviral encoding a scramble control, shUCP2#2, or shUCP2#3 plasmid for 72 h. GFP-positive cells were sorted using flow cytometry. Stable transfectants were sub-cultured before being subjected to **(A)** cell viability and **(B)** cell cycle determination by flow cytometry. **(C)** Cell number was counted using cell countess and normalized to the scramble control. Oxidative stress indexes, including **(D)** ATP, **(E)** NADP/NADPH ratio, and **(F)** GSH/GSSG ratio, were determined according to the manufacturer’s instructions. **(G)** Mitochondrial ROS, **(H)** mitochondrial mass, and **(I)** mitochondrial membrane potential were determined according to the instruction manual. **(J)** Mitochondrial morphology (indicated by red box) was determined using high-power electron microscopy, the number of mitochondrial fusions was quantified using Image J software, represented as mitochondria number/mm, and data were normalized to the scramble control. Mitochondria morphology was indicated in the red box. UCP2 silencing THP-1 AML xenografts were generated as described, and mice were sacrificed 12 days post-transplantation. Tumorigenesis-related indexes, including **(K)** mouse body weight, **(L)** survival, **(M)** cell viability, **(N)** the size of spleen and liver, **(O)** AML engraftment (hCD45^+^), and **(P)** mitochondrial ROS from bone marrow mononuclear cells, were determined using flow cytometry. All experiments were repeated three times. Data represent mean ± standard deviation from technical triplicates (∗*p* < 0.01, ∗∗*p* < 0.05, and ∗∗∗*p* < 0.001; two-way ANOVA for scramble control versus shUCP2s). UCP2, uncoupling protein 2; AML, acute myeloid leukemia; NADP, nicotinamide adenine dinucleotide phosphate; NADPH, reduced NADP; GSH, reduced glutathione; GSSG, oxidized glutathione; ROS, reactive oxygen species.

Despite the mounting data indicating the diverse cellular roles of UCP2 in various solid tumors, its molecular role in hematological malignancy remains controversial. We set up to explore the biological effects of UCP2 on AML cells. To knock down the endogenous UCP2 protein, we generated THP-1 or KG-1 cells with stable transfectants either overexpressing a lentiviral encoding a scramble control, shUCP2#1, shUCP2#2, or shUCP2#3 plasmid. As illustrated in Figure S1, endogenous UCP2 protein and transcription levels were significantly abolished with shUCP2#2 & shUCP2#3. As a result, silencing UCP2 promoted leukemic cell apoptosis, induced G_1_/S arrest, and reduced cell growth (Fig. 3A–C). These data suggest the oncogenic role of UCP2 in leukemogenesis. Since UCP2 is well known to maintain mitochondrial homeostasis via modulating ROS and proton transmission across the mitochondrial double membrane, we then explored the effects of UCP2 on mitochondrial function. Silencing UCP2 reduced ATP production, concordant with up-regulated NADP/NADPH ratio and down-regulated GSH/GSSG ratio (Fig. 3D–F). Moreover, silencing UCP2 elevated mitochondrial ROS, accompanied by increased mitochondrial mass and mitochondrial membrane potential (Fig. 3G–I). Furthermore, Figure 3J demonstrated that silencing UCP2 also resulted in swelling of AML cells and increased numbers of mitochondrial fusions. These results suggest that inhibition of UCP2 caused an imbalance in mitochondrial homeostasis. To further explore the biological significance of UCP2 on leukemic cells *in vivo.* Two million shUCP2#2 and shUCP2#3 lentivirus-transduced THP1 cells were intravenously injected into each NOD/SCID mouse (*n* = 5). Eleven days post-transplantation, the AML xenograft mice were sacrificed, and tumorigenesis-related phenotypes were measured. Although there was no significant difference in mouse body weight, overall survival was increased in UCP2 knockdown AML xenografts (Fig. 3K and L), and the log-rank (Mantel–Cox) test was used for survival analysis. In addition, decreased cell viability was in concordance with smaller-sized spleen and liver as well as eradicated AML engraftment in shUCP2 AML xenografts (Fig. 3M−O). Furthermore, mitochondrial ROS were also elevated upon silencing UCP2 (Fig. 3P). These data indicate that silencing UCP2 inhibits leukemogenesis *in vivo,* and UCP2 could be a potential therapeutic target to improve AML treatment.

### Silencing UCP2 elevated BCAA levels in AML

**Figure 4:**
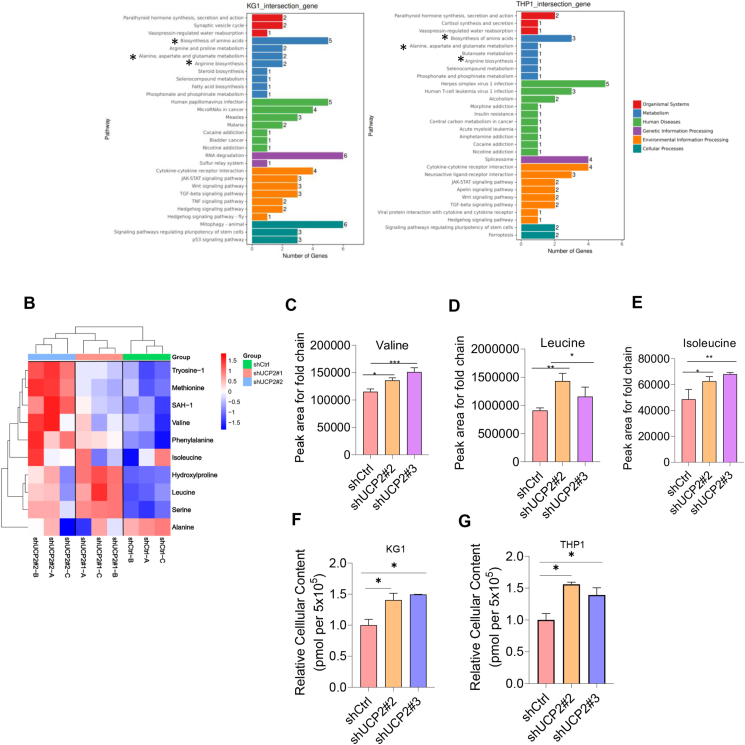
Genetically knockdown of UCP2 up-regulated BCAA in acute myeloid leukemia. THP-1 or KG-1 cells overexpressing either a scramble control, shUCP2#2, or shUCP2#3 stable transfectants were subjected to RNA-sequencing analysis. **(A)** KEGG pathway analysis of the top signaling pathways that were aberrantly regulated in the above stable transfectants (∗common signaling pathways in both cell lines). THP-1 or KG-1 cells overexpressing either a scramble control, shUCP2#2, or shUCP2#3 stable transfectants were subjected to metabolic mass spectrometry. **(B)** Heatmap of the expression profile of amino acid metabolism-related gene expression. **(C**–**E)** The peak area for the fold chain of amino acid gene expression (data were normalized to scramble control). **(F, G)** The BCAA content of THP-1 or KG-1 cells overexpressing either a scramble control, shUCP2#2, or shUCP2#3 stable transfectants (data were normalized to the scramble control). All experiments were repeated three times. Data represent mean ± standard deviation from technical triplicates (∗*p* < 0.01, ∗∗*p* < 0.05, ∗∗∗*p* < 0.001, and ∗∗∗∗*p* < 0.005; two-way ANOVA for scramble control versus shUCP2s). BCAA, branched-chain amino acid; UCP2, uncoupling protein 2.

To elucidate the molecular mechanism underlying UCP2 promoting leukemogenesis, we performed transcription sequencing analysis in KG-1 and THP1 cells overexpressing lentiviral encoding a scramble control, shUCP2#2, or shUCP2#3 plasmid. As illustrated in Figure 4A, RNA-sequencing analysis revealed that amino acid metabolism was aberrantly expressed upon UCP2 knockdown in both cell lines. We then performed metabolic mass spectrometry to validate whether silencing UCP2 affected amino acid metabolism (Fig. 4B). Indeed, leucine/valine/isoleucine levels were up-regulated in the UCP2 knockdown group in comparison to the scramble control (Fig. 4C–E), and suppression of UCP2 also enhanced cellular content of BCAA in AML (Fig. 4F and G). These data suggest that BCAA is a potential downstream target of UCP2 in AML.

### Accumulation of BCAA-induced oxidative stress activates the PI3K/AKT/mTOR signaling pathway in AML

**Figure 5:**
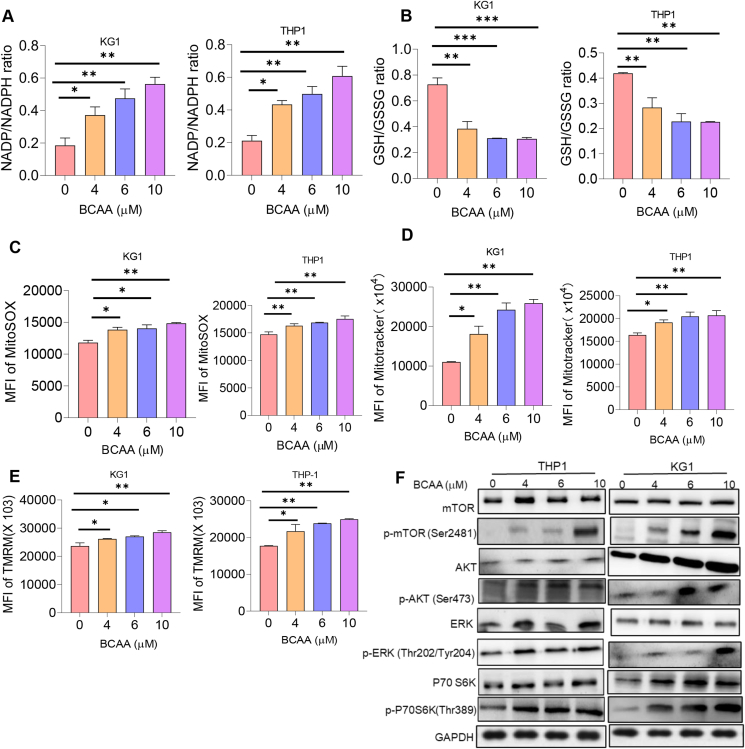
BCAA accumulation-induced oxidative stress activates the PI3K/AKT/mTOR signaling pathway in acute myeloid leukemia. THP-1 and KG-1 cells, two acute myeloid leukemia cell lines that exhibits high level of endogenous UCP2, were treated with increasing doses of BCAA (4 μM, 6 μM, and 10 μM) for 24 h before determination of **(A)** NADP/NADPH ratio, **(B)** GSH/GSSG ratio, **(C)** mitochondrial ROS, **(D)** mitochondrial mass, and **(E)** mitochondrial membrane potential. **(F)** Immunoblotting was performed to determine the expression of PI3K/AKT/mTOR signaling proteins. All experiments were repeated three times. Data represent mean ± standard deviation from technical triplicates (∗*p* < 0.01, ∗∗*p* < 0.05, ∗∗∗*p* < 0.001, and ∗∗∗∗*p* < 0.005; two-way ANOVA for the untreated versus the treated. NADP, nicotinamide adenine dinucleotide phosphate; NADPH, reduced NADP; GSH, reduced glutathione; GSSG, oxidized glutathione; ROS, reactive oxygen species; BCAA, branched-chain amino acid; UCP2, uncoupling protein 2.

To investigate whether the high level of BCAA triggered oxidative stress in AML cells, we treated THP-1 and KG1 cells with increased doses of BCAA as indicated. As a result, an increased concentration of BCAA up-regulated the NADP/NADPH ratio, indicating enhanced NADPH oxidation, whereas the GSSG/GSH ratio was significantly reduced (Fig. 5A and B). In addition, mitochondrial ROS was elevated in response to high levels of BCAA (Fig. 5C). Furthermore, mitochondrial mass and mitochondrial membrane potential were also increased upon accumulation of BCAA (Fig. 5D and E). These data suggest that a high level of BCAA is attributed to oxidative stress. A previous study reported that BCAA modulated metabolic and cellular activity via activating the PI3K/AKT/mTOR signaling pathway to promote the activity of circulating peripheral blood mononuclear cells,^31^ and thus, we further performed immunoblotting to explore whether high levels of BCAA induced oxidative stress in AML via regulating the PI3K/AKT/mTOR signaling pathway. As demonstrated in Figure 5F, the p-AKT, p-ERK, mTOR, and p-S6K protein levels were significantly elevated in response to high levels of BCAA. These data imply that a high level of BCAA induced oxidative stress via activating the PI3K/AKT/mTOR signaling pathway in AML.

### Lack of BCAA restored UCP2 silencing-induced anti-leukemogenesis and oxidative stress phenotypes

**Figure 6:**
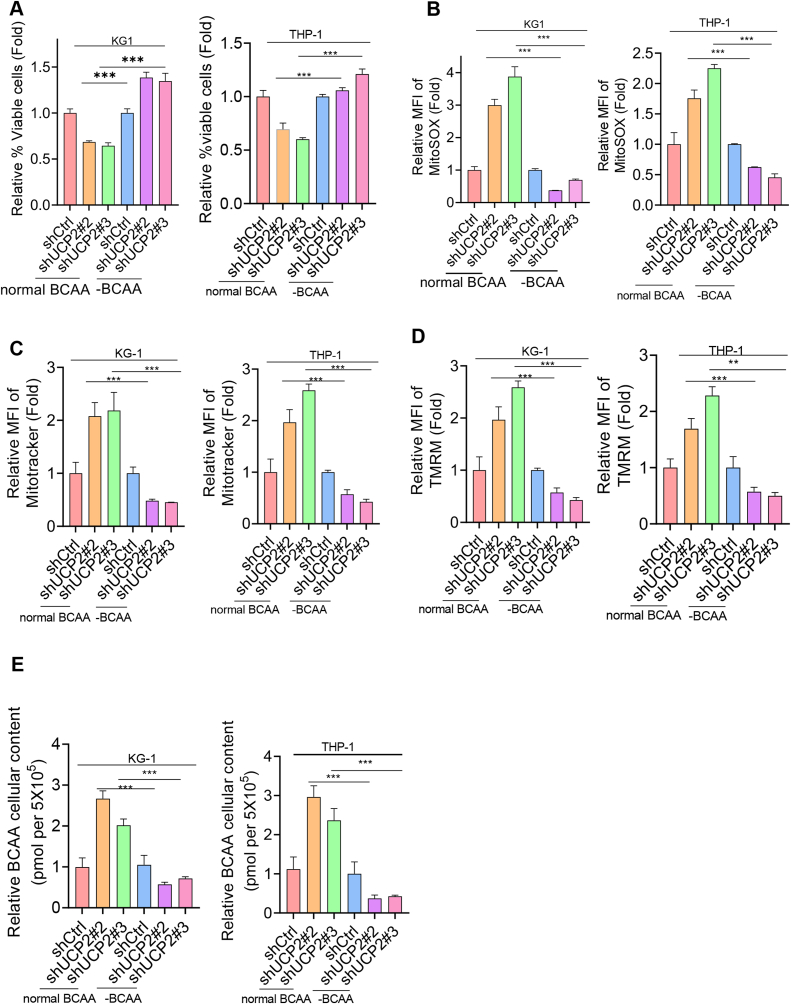
Lack of BCAA restored UCP2 silencing-induced anti-leukemogenesis and oxidative stress phenotypes. THP-1 or KG-1 cells overexpressing either a scramble control, shUCP2#2, or shUCP2#3 stable transfectants in the proliferating phase were sub-cultured either in a normal or low level of BCAA as described previously before being subject to the following experiments. **(A)** Cell viability or metabolic-related assays, including **(B)** mitochondrial ROS, **(C)** mitochondrial mass, and **(D)** mitochondrial membrane potential, were determined using flow cytometry. **(E)** BCAA levels were measured according to the instruction manual. All data were represented as fold change (normalized to shCtrl), and all experiments were repeated three times. Data represent mean ± standard deviation from technical triplicates (∗*p* < 0.01, ∗∗*p* < 0.05, ∗∗∗*p* < 0.001, and ∗∗∗∗*p* < 0.005; two-way ANOVA for normal BCAA versus lack of BCAA). ROS, reactive oxygen species; BCAA, branched-chain amino acid; UCP2, uncoupling protein 2.

Since our omics data have demonstrated that silencing UCP2-induced oxidative stress is strongly associated with BCAA in AML, we then investigated whether the lack of BCAA restored UCP2 silencing-induced phenotypes. We sub-cultured THP-1 and KG-1 cells overexpressing lentiviral encoding shUCP2#2 or shUCP2#3 lentiviral plasmid in culture medium supplemented either with or without BCAA, respectively. As a result, leukemic cell survival was increased in the low-level-BCAA group compared with the normal BCAA group (Fig. 6A). In addition, mitochondrial ROS, mitochondrial mass, and mitochondrial membrane potential were also decreased while lacking BCAA (Fig. 6B–D). Moreover, the cellular content of BCAA was also reduced while lacking BCAA (Fig. 6E). These results further suggest that BCAA is a potential regulatory target of UCP2 in AML.

### Genipin induces leukemic cell death by accumulating BCAA

**Figure 7:**
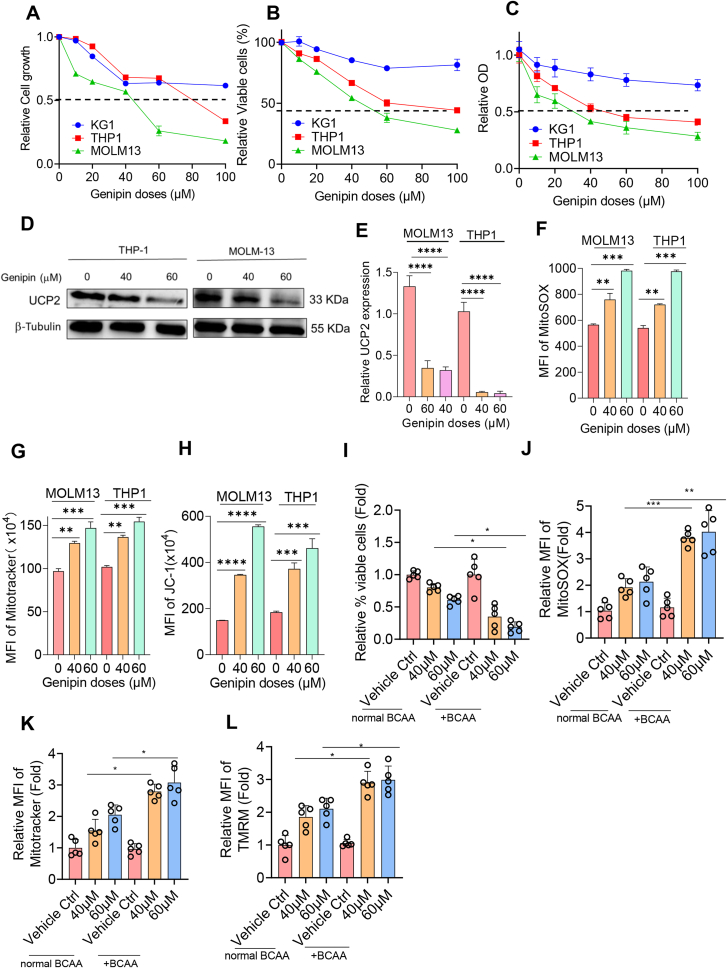
Genipin induced leukemic cell death via accumulating BCAA. KG-1, THP-1, and MOLM-13 cells were treated either with or without genipin at the indicated doses (20 μM, 40 μM, 60 μM, 80 μM, and 100 μM) for 48 h before determination of **(A)** cell growth using cell countess, **(B)** cell viability using flow cytometry, and **(C)** cell cytotoxicity using CCK8 assay. Data were normalized to the scramble control. UCP2 protein level and mRNA level were determined either using **(D)** immunoblotting or **(E)** quantitative PCR upon genipin treatment. Metabolism-related assays, including **(F)** mitochondrial ROS, **(G)** mitochondrial mass, and **(H)** mitochondrial membrane potential, were determined using flow cytometry. Five *de novo* acute myeloid leukemia primary cells were sub-cultured either in the normal or high level of BCAA culture medium before being treated either with 40 μM or 60 μM genipin for 48 h, respectively. Primary cells were then subjected to **(I)** cell viability or **(J**–**L)** mitochondria-related assays (data were normalized to the vehicle control). All experiments were repeated three times. Data represent mean ± standard deviation from technical triplicates (∗*p* < 0.01; ∗∗*p* < 0.05; ∗∗∗*p* < 0.001, and ∗∗∗∗*p* < 0.005; two-way ANOVA for the untreated versus the treated, and normal BCAA versus high BCAA). ROS, reactive oxygen species; BCAA, branched-chain amino acid; UCP2, uncoupling protein 2.

To elucidate whether inhibition of UCP2 eliminated leukemic cells, we utilized genipin, a selective inhibitor that pharmacologically targets UCP2, to treat either THP-1 or MOLM-13 cells that exhibit high levels of endogenous UCP2. As demonstrated in Figure 7A–C, genipin inhibited leukemic cell growth and induced apoptosis and cytotoxicity to leukemic cells. In addition, genipin reduced the protein and transcription level of UCP2 in both THP-1 and MOLM-13 cells (Fig. 7D and E). Interestingly, genipin enhanced mitochondrial ROS production, and concomitantly increased mitochondrial mass and mitochondrial membrane potential (Fig. 7F–H). To further confirm whether genipin induced leukemic cell death and oxidative stress via enhancing BCAA level, we sub-cultured five *de novo* AML primary cells (see Table S1 for patients’ clinical information) either with normal or high levels of BCAA as described previously, followed by treatment either with 40 μM or 60 μM genipin for 48 h, respectively. Interestingly, a high level of BCAA induced more leukemic cell death, enhanced NADPH oxidase, and elevated mitochondrial mass and mitochondrial membrane potential, compared with the normal BCAA group (Fig. 7I–L). These results indicate that a high level of BCAA plays an essential role in maintaining knockdown UCP2-induced leukemic cell death.

### Supplementation of BCAA enhances the anti-tumor efficacy of genipin

**Figure 8:**
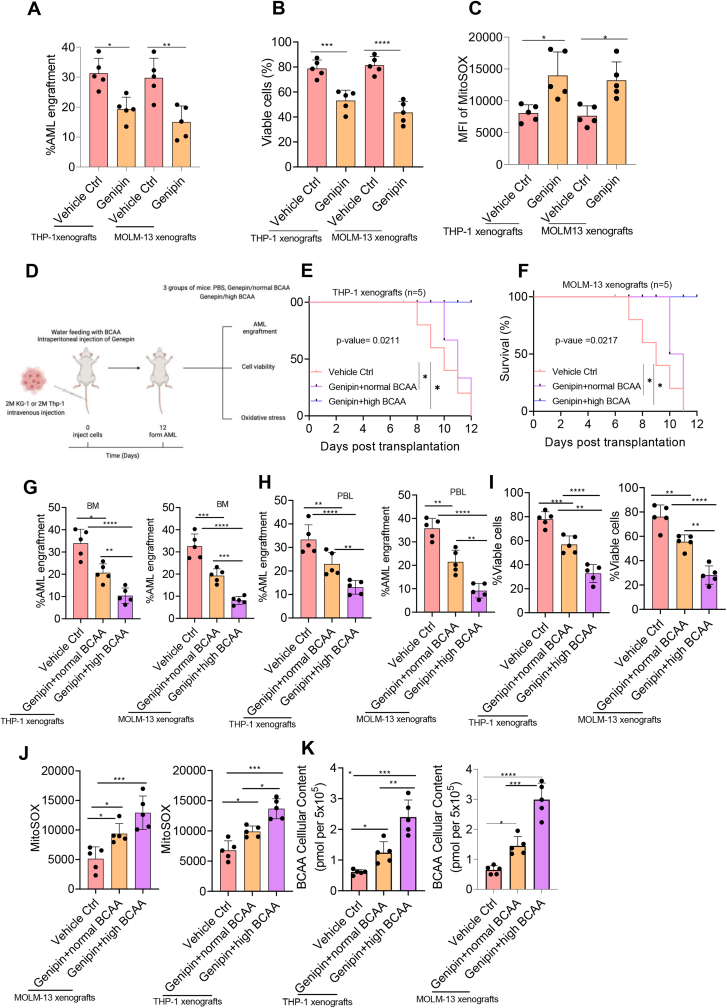
Supplementation of BCAA enhanced the anti-tumor activity of genipin. Two million THP-1 or MOLM-13 cells were transplanted into six-week-old nude mice via the tail vein injection; meanwhile, mice were administered either PBS (vehicle Ctrl) or 25 mg/kg genipin every two days via intraperitoneal injection. Mice were sacrificed, and tumorigenesis-related indexes, including **(A)** percentage of AML engraftment, **(B)** cell viability, and **(C)** mitochondrial ROS, were assessed. **(D)** Mice were treated with either PBS (vehicle Ctrl) or 25 mg/kg genipin as described previously. In the high BCAA group, mice were fed with water containing 15 mg/mL valine, 15 mg/mL leucine, and 15 mg/mL isoleucine throughout the entire experiment. In the normal BCAA group, mice were fed with normal sterile ddH_2_O. Mice were sacrificed 12 days post-transplantation. Tumorigenesis-related indexes, including **(E, F)** mice survival, **(G, H)** percentage of AML engraftment in bone marrow (BM) and peripheral blood (PBL), **(I)** cell viability, and **(J)** mitochondrial ROS, were assessed. **(K)** The BCAA level was also determined upon withdrawing mouse blood via the posterior ophthalmic venous plexus. The survival analysis was performed using the log-rank (Mantel–Cox) test. All experiments were repeated three times. Data represent mean ± standard deviation from technical triplicates (∗*p* < 0.01, ∗∗*p* < 0.05, ∗∗∗*p* < 0.001, and ∗∗∗∗*p* < 0.005; two-way ANOVA for genipin plus normal BCAA versus genipin plus high BCAA. AML, acute myeloid leukemia; ROS, reactive oxygen species; BCAA, branched-chain amino acid.

To elucidate whether pharmacological inhibition of UCP2 eradicated AML blasts and prolonged mice survival, we administered UCP2 high AML xenografts derived either from THP-1 or MOLM-13 cell lines, with 25 mg/kg genipin every two days via intraperitoneal injection. Twelve days post-transplantation, mice were sacrificed and analyzed. Indeed, pharmacological inhibition of UCP2 significantly eradicated AML blasts, induced leukemic cell death, and enhanced mitochondrial ROS production compared with vehicle control (Fig. 8A–C). These results indicate that UCP2 could be a potential therapeutic target in abrogating leukemogenesis. Since we implied that genipin induced AML apoptosis through accumulating BCAA *in vitro*, we then set out to explore whether supplementation of BCAA enhanced the anti-tumor activity of genipin; we administered mice either with PBS (vehicle control) or 25 mg/kg genipin every two days via intraperitoneal injection, and meanwhile, fed them either with normal BCAA or high BCAA diet throughout the entire procedure as described. Interestingly, genipin/high BCAA mice group exhibited a longer survival rate, and dramatically reduced AML engraftment in both peripheral blood and bone marrow (Fig. 8D–H). In addition, supplementation of BCAA alongside genipin eliminated more leukemic cells compared with the normal BCAA group (Fig. 8I). Furthermore, the BCAA high group resulted in the accumulation of mitochondrial ROS and BCAA in the plasma in contrast to the normal BCAA diet (Fig. 8J and K). These results elucidate the potential therapeutic application of BCAA metabolism against hematological malignancy.

## Discussion

In summary, our present study elucidated that UCP2 was highly activated in high-risk AML groups and was significantly associated with poor prognosis and therapy resistance. Silencing UCP2 increased BCAA and thus abrogated leukemogenesis. In addition, supplementation of BCAA enhanced the anti-tumor activity of genipin, a selective inhibitor of UCP2 in AML xenografts (Fig. S2). Taken together, these data indicate that suppression of UCP2 induces leukemic cell death through accumulation of BCAA, and targeting BCAA in AML could be an effective therapeutic approach to improve the clinical outcome of AML treatment.

Mitochondrial dysfunction has been increasingly recognized as a key player in the pathogenesis of leukemia. For instance, leukemic cells favor oxidative phosphorylation rather than glycolysis to survive.^32^ In addition, metabolic reprogramming is a key intrinsic mechanism that enhances the survival of AML cells and contributes to their resistance to therapeutic agents.^33^ Our study revealed that metabolism-related genes were significantly up-regulated in the high-risk AML group, and associated with poor AML prognosis, in which UCP2 conferred therapy resistance in AML. This implies potential therapeutic application in targeting metabolic proteins in hematological malignancies. Our findings are consistent with others and demonstrate the multifaceted roles of UCP2 in shielding cells from oxidative damage and modulating tumor growth through alterations in glycolysis and oxidative metabolism.^34^

Mounting evidence has demonstrated the oncogenic role of UCP2 in promoting tumor cell proliferation and tumorigenesis via modulating mitochondrial function. For instance, UCP2 maintains leukemic cell survival via remodeling glutamine and promoting non-small cell lung cancer growth by switching cells to glycolysis,^15^ promotes pancreatic ductal adenocarcinoma cell proliferation via reducing ROS production,^35^ and acts as a potential metabolic biomarker in colon carcinogenesis.^36^ In addition, in acute lymphoblastic leukemia, UCP2 modulates mitochondrial function by regulating glutamine uptake, which is essential for their growth and proliferation,^37^ and its effects on the metabolism of glutamine and other nutrient impacts cellular energy production via glutaminolysis and redox balance in acute lymphoblastic leukemia,^37^ thus contributing to cell stemness and survival ability. The mechanisms underlying UCP2-modulated metabolic reprogramming in hematological malignancies remain unclaimed and under-interpreted. Our multi-omics study revealed that suppression of UCP2 elevated BCAA in AML, and lack of BCAA restored silencing UCP2-induced phenotypes, which suggests that UCP2 is an upstream regulator of BCAA-induced oxidative stress in AML. Despite others have reported that branched chain amino acid transaminase 1 (BCAT1), a cytosolic aminotransferase for BCAA, is up-regulated during the progression of chronic myeloid leukemia and aberrantly expressed in enhancer of zeste homolog 2 (EZH2)-deficient myeloid neoplasia, promotes BCAA production in leukemia cells, and maintains leukemia cell stemness,^38^, ^39^, ^40^ we revealed that elevated BCAA metabolism trigged leukemic cell death under the circumstances of silencing UCP2-induced metabolic dysfunction, which is in concordance with previous reports that accumulation of BCAA is strongly associated with oxidative stress.^41^ The mechanism of silencing UCP2-enhanced BCAA is not fully addressed by us, and one possible reason could be due to impaired enzyme activity in BCAA catabolism, as a previous study reported that decreased BCKD activity was the main cause of increased BCAA levels in diabetes type 2 obesity.^41^

The targeted therapeutic role of BCAA metabolism in cancer treatment has been implicated in other tumor types. For instance, BCAT1 confers epidermal growth factor receptor (EGFR)-tyrosine kinase inhibitor (TKI) resistance through epigenetic glycolytic activation in non-small cell lung cancer^42^; BCAT2-mediated BCAA catabolism is essential for the development of pancreatic ductal adenocarcinoma^43^; and BCAA-producing *Clostridium symbiosum* promotes colorectal tumorigenesis.^44^ The impact of BCAA-induced oxidative stress in hematological malignancy is not well understood. Our *in vitro* data demonstrated that a high level of BCAA resulted in oxidative stress via activating PI3K/AKT/mTOR, which has been recognized to be associated with various cellular events, including cell growth, differentiation, and stemness.^45^ However, the AML engraftments also demonstrated that silencing or inhibition of UCP2 using a natural selective inhibitor (genipin) eradicated leukemic cells and reduced the mice’s survival, and supplementation of BCAA enhanced the anti-leukemogenesis effects of genipin, further implying the significance of UCP2 in mediating BCAA accumulation-induced oxidative stress and metabolic changes in leukemogenesis. Based on these findings, we conclude that inhibition of UCP2 can eradicate leukemic blasts by up-regulating BCAA-induced oxidative stress, making it suitable for potential application in AML treatment.

In conclusion, our study shed light on the potential prognostic role of UCP2 in AML, the impacts of UCP2-mediated BCAA metabolism in leukemogenesis, and the future direction of targeting metabolic proteins or their mediated signaling pathway in improving the clinical outcomes of hematological malignancies.

## CRediT authorship contribution statement

**Agida Okohi Innocent:** Formal analysis, Investigation, Methodology, Data curation. **Yajie Shen:** Methodology, Data curation, Formal analysis, Investigation. **Yixuan Gao:** Software, Visualization. **Ruixin Sun:** Data curation, Formal analysis. **Kasimujiang aximujiang:** Writing – review & editing. **Zizhen Xu:** Software, Data curation, Validation, Formal analysis, Resources. **Jinke Cheng:** Funding acquisition, Conceptualization. **Jiao Ma:** Supervision, Writing – original draft, Conceptualization, Funding acquisition.

## Ethics declaration

Primary AML cells were obtained from the Department of Hematology at Ruijin Hospital Affiliated to Shanghai Jiao Tong University, School of Medicine after Institutional Review, Board review and approval (2011–06). All mouse experiments were performed under an Institutional Animal Care and Use Committee-approved protocol, and institutional guidelines for the proper use of animals in research were followed (JUMC2023-127-A).

## Data availability

RNA-sequencing data were deposited in the GEO database (accession numbers: GSE284102 and GSE285525).

## Conflict of interests

There are no competing financial interests to declare.
