# Supplementary material for: Suppression of UCP2 alleviates leukemogenesis by enhancing branched-chain amino acids-induced oxidative stress via activating the PI3K/AKT/mTOR signaling pathway
Source: Genes Dis. 2025 Aug 5;13(4):101794. doi: 10.1016/j.gendis.2025.101794 (PMC13089170; doi:10.1016/j.gendis.2025.101794)
Supplement: Multimedia component 1 [file mmc1.pdf]

ID	Treatment regimen	Clinical outcome	FAB classification	Gender	Age
AML1	hyperCVAD-A+venetoclax	CD	ETP-ALL	M	34
AML2	IA	CR	M4	F	38
AML3	IA	CR	AML	M	33
AML4	Aza+Ven，transplant	CR	MDS transformed AML	F	30
AML5	N/A	N/A	M4	M	51
AML6	Aza+Ven+IDA	MLFS	M4	M	63
AML7	CMG+VEN	CR	M5	F	55
AML8	Aza+Ven	relapse	M5	F	61
AML9	N/A	N/A	M2	M	65
AML10	ATRA+ATO+IDA	CR	M4	M	35
AML11	ATRA+ATO	Relapse	M3	F	37
AML12	mini-CVD+VEN	Relapse	ALL-L2，T-ALL	M	74
AML13	IA	Relapse	M4	M	28
AML14	IA	Relapse	M4	F	26
AML15	IDA+Aza+Ven	Relpase	M5	M	60
AML16	HA	Relapse	M4	M	9
AML17	IA	Relpase	M4-Eo	M	41
AML18	IA	Relapse	M5	M	47
AML19	ATRA+ATO	Relapse	M3	F	59
AML20	Aza+Ven,CLAGE	Relapse	MDS IB2	F	23
AML21	IDA+Aza+Ven	Relapse	M4	M	62
AML22	ATRA+ATO+IDA	Relpase	M3	M	40
AML23	low dose Ara-C	Relapse	M4	F	19
AML24	IDA+Aza+Ven	Relapse	M5	M	17
AML25	IDA+Aza+Ven	Relapse	AML-M5b	F	61
AML26	Aza+Ven	Relapse	AML-MR	M	72
AML27	IA	Relapse	M5	M	47
AML28	Aza+Ven	Relapse	M5	F	61

Table S1: AML primary cell clinical information.

NB: AML1-AML10 primary cells were used to determine the protein levels of UCP2 (please refer to Figure 1E); AML1-AML5 primary cells were also used for ex vivo genipin treatment (please refer to Figure 7I-7L); AML11-AML28 relapse and paired de novo were used to determine the mRNA level of UCP2 (please refer to Figure 1F).

Figure S1

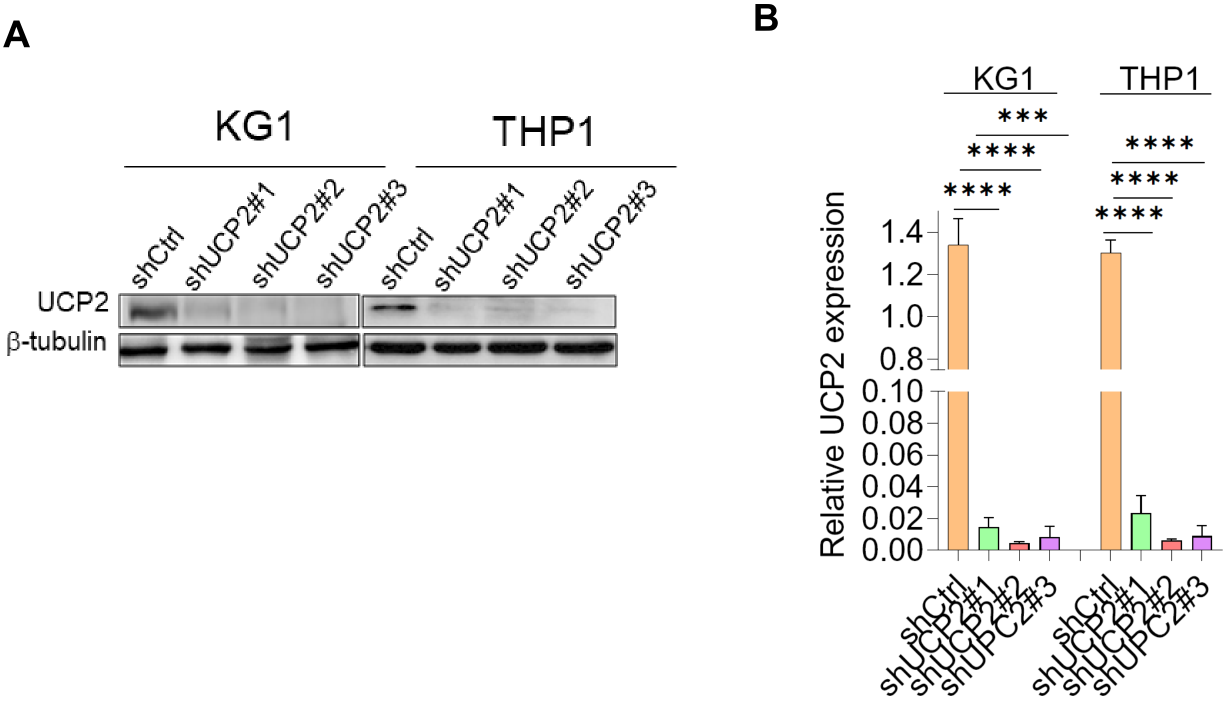

Figure S1. Knockdown efficiency of shUCP2#1, shUCP2#2 and shUCP2#3 in KG-1 and THP-1 cells. Lentiviral encoding shCtrl, shUCP2#1, shUCP2#2 plasmids were transduced in KG-1 or THP-1 cells for 72 h, respectively, followed by flow cytometry sorting of GFP+ cells to obtain stable transfectants, which then subjected either to (A) Immunoblotting or (B) qPCR. All experiments were repeated three times, and graph with error bars show the data represent the mean + SD from technical triplicates (****$p<0.005$), compared shUCP2#2 and shUCP2#3 to shCtrl.

Figure S2

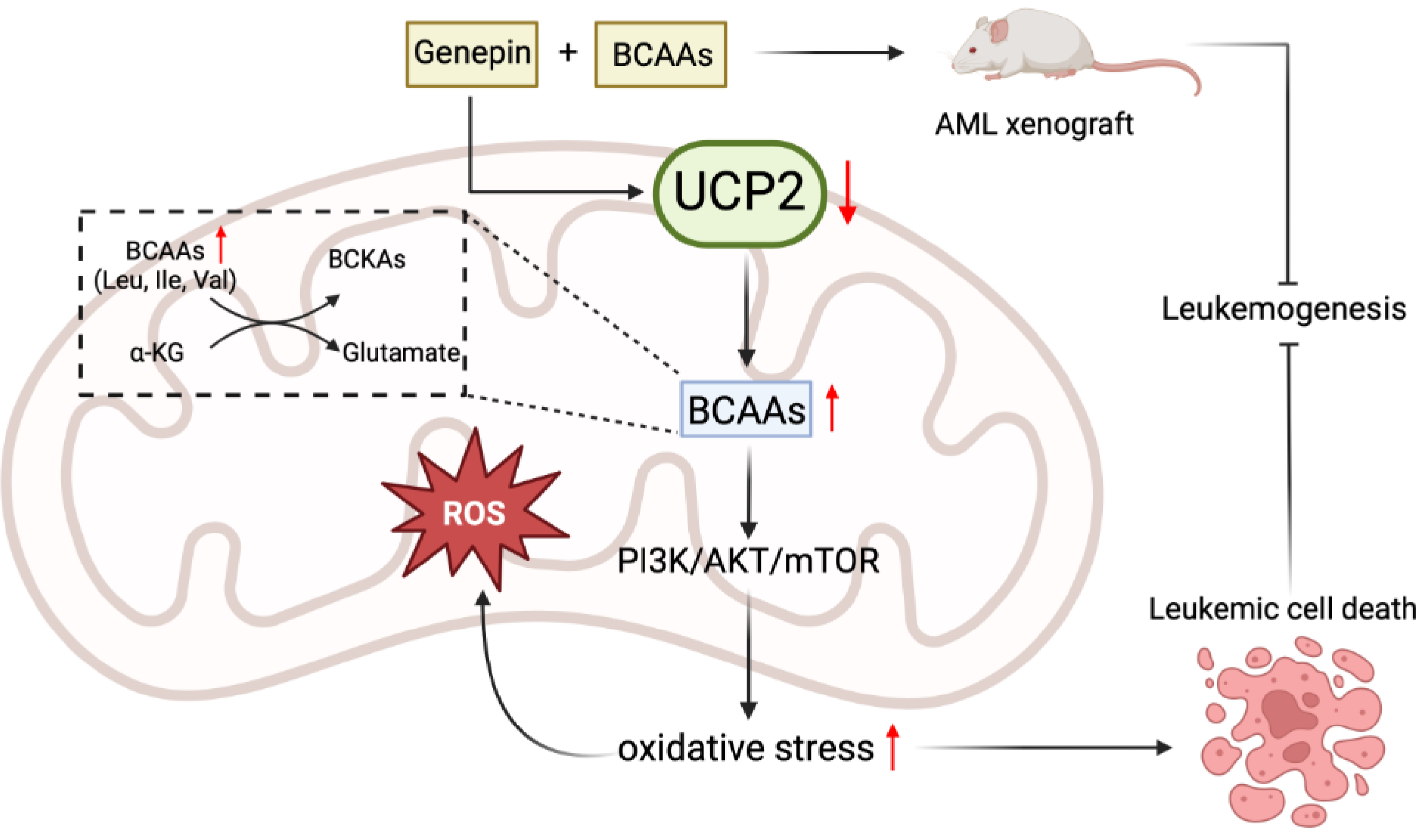

Figure S2. Graphic illustration of the mechanisms of suppression of UCP2 in alleviating leukemogenesis. Silencing UCP2 upregulates BCAA through activation of PI3K/AKT/mTOR signaling pathway to abrogate leukemogenesis. In addition, supplementation of BCAA enhanced the anti-tumor activity of Genipin, a selective inhibitor of UCP2 in AML xenografts.
